# Fully Additively Manufactured Counter Electrodes for Dye-Sensitized Solar Cells

**DOI:** 10.3390/mi15040464

**Published:** 2024-03-29

**Authors:** Semih Akin, Sungdo Kim, Chul Ki Song, Sang Yong Nam, Martin Byung-Guk Jun

**Affiliations:** 1 Department of Mechanical, Aerospace and Nuclear Engineering, Rensselaer Polytechnic Institute, Troy, NY 12180, USA; 2 School of Mechanical Engineering, Purdue University, West Lafayette, IN 47907, USA; kimsde97@naver.com; 3 Department of Mechanical Engineering, Gyeongsang National University, Jinju 52828, Republic of Korea; 4 School of Mechanical Engineering, ERI, Gyeongsang National University, Jinju 52828, Republic of Korea; cksong@gnu.ac.kr; 5 Department of Materials Engineering and Convergence Technology, Gyeongsang National University, Jinju 52828, Republic of Korea; walden@gnu.ac.kr

**Keywords:** dye-sensitized solar cells (DSSC), counter electrode, additive manufacturing, 3D printing, cold spray, polymer metallization

## Abstract

In dye-sensitized solar cells (DSSCs), the counter electrode (CE) plays a crucial role as an electron transfer agent and regenerator of the redox couple. Unlike conventional CEs that are generally made of glass-based substrates (e.g., FTO/glass), polymer substrates appear to be emerging candidates, owing to their intrinsic properties of lightweight, high durability, and low cost. Despite great promise, current manufacturing methods of CEs on polymeric substrates suffer from serious limitations, including low conductivity, scalability, process complexity, and the need for dedicated vacuum equipment. In the present study, we employ and evaluate a fully additive manufacturing route that can enable the fabrication of CEs for DSSCs in a high-throughput and eco-friendly manner with improved performance. The proposed approach sequentially comprises: (1) material extrusion 3-D printing of polymer substrate; (2) conductive surface metallization through cold spray particle deposition; and (3) over-coating of a thin-layer catalyzer with a graphite pencil. The fabricated electrodes are characterized in terms of microstructure, electrical conductivity, and photo-conversion efficiency. Owing to its promising electrical conductivity (8.5 × 10^4^ S·m^−1^) and micro-rough surface structure (R_a_ ≈ 6.32 µm), the DSSCs with the additively manufactured CEs led to ≈2.5-times-higher photo-conversion efficiency than that of traditional CEs made of FTO/glass. The results of the study suggest that the proposed additive manufacturing approach can advance the field of DSSCs by addressing the limitations of conventional CE manufacturing platforms.

## 1. Introduction

Renewable energy technologies are of particular interest to solving the global energy crisis. Among others, solar energy emerges as the most promising and viable energy source owing to its abundance, low cost, and safety [1]. To harvest solar energy, photovoltaic (PV) technology involving solar cells plays a pivotal role in harnessing solar energy, converting it into electric energy. Solar cells are categorized into three main generations: (i) first-generation solar cells made of crystalline silicon; (ii) second-generation solar cells, commonly referred as thin-film solar cells: and (iii) third generation solar cells, which are based on novel materials [2]. First-generation silicon-based solar cells present challenges due to their rigid, fragile, and low-absorption properties. Second generation thin-film solar cells suffer from high costs and a scarcity of active materials, long payback, and poor performance in cloudy and shadowy conditions [3,4]. Third-generation solar cells are considered to be a pragmatic replacement for both first and second generation solar cells by comprising novel materials such as organic dyes, conducting polymers, and nanostructured materials. Among third-generation solar cells, dye-sensitized solar cells (DSSCs) have gained considerable attention owing to their inherent advantages, including low material cost, tunable optical properties, flexibility, and good performance in a wide range of lighting conditions [5,6]. Furthermore, recent advancements in bifacial DSSC technology have attracted considerable attention, enabling the absorption of sunlight from both front and rear sides [7].

**Figure 1:**
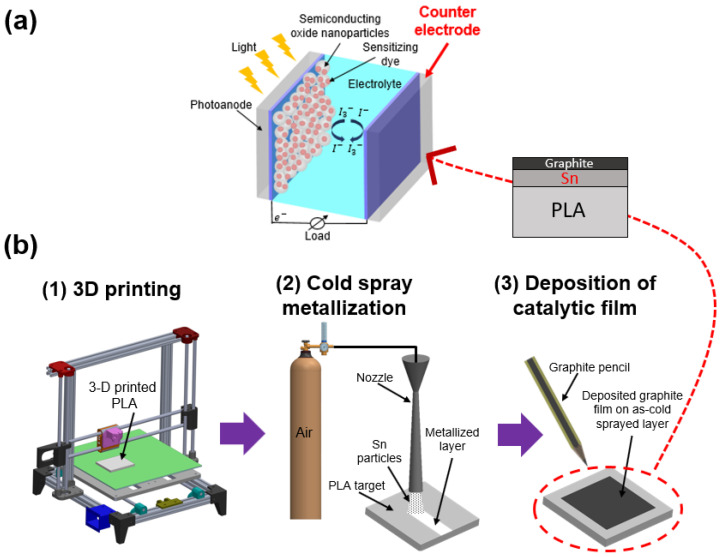
Schematic of (**a**) a typical DSSC; (**b**) proposed additive manufacturing approach.

A typical DSSC, as shown in Figure 1a, is mainly composed of a photoanode (i.e., contains a dye-sensitized porous semiconductor such as titanium dioxide (TiO_2_)), an electrolyte, and a counter electrode. In the energy conversion process, first, light is absorbed by the sensitizer (i.e., dye molecules on a TiO_2_ layer of the photoanode). This photoexcitation generates excited electrons, which are then injected into the conduction band of the semiconductor. The injected electrons further travel through the semiconductor towards the photoanode, and then reach the counter electrode (CE). CEs act as a catalyst and inject the electrons into the electrolyte (i.e., iodine/tri-iodine redox couple) by reducing the electrolyte. Lastly, the electrons reach the dye molecules again due to the diffusion process, completing a cyclic circuit [3,8,9].

As for DSSC architecture, the CE—which is a conductive substrate with a thin layer of catalytic film on it—plays a critical role in completing the circuit by acting as the catalyst for reducing the electrolyte to regenerate dye molecules [4]. As such, the CE is a critical component in DSSCs, improving the efficiency of the cell [10]. A suitable CE should possess a high electrical conductivity, large surface area, porous surface nature, excellent stability, catalytic activity, and good mechanical adhesion for achieving remarkable photo-conversion efficiency [5,11]. Traditional CEs are made of glass-based substrates coated with indium–tin oxide (ITO) or fluorine-doped tin oxide (FTO) [5]. These materials, however, suffer from brittleness, fragility, heavy weight, poor electrical conductivity, high-manufacturing costs, and the need for high-temperature treatment [6,12], thereby limiting the large-scale deployment of DSSCs. Furthermore, the catalyst materials deposited on these conductive oxides to constitute the CE are crucial for enhanced performance. One of the most common catalyst materials in DSSCs is platinum (Pt) due to its remarkable catalytic activity and high electrical conductivity [13]. However, Pt is prohibitively expensive, rare, and sensitive to contaminants [7], thereby limiting its widespread use in DSSCs. As such, the pragmatic deployment of DSSCs relies on the utilization of durable, recyclable, low-cost substrates with abundant and low-cost catalyst materials.

Recently, the deposition of electrodes on polymer substrates has emerged as a promising alternative to traditional CEs in DSSCs. This approach offers increased versatility owing to the lightweight, abundance, low-cost, porous nature, and impact resistance of polymer substrates [4]. Conventional methods for producing polymer-based CEs primarily involve vapor deposition, sputter coating, electrochemical deposition, or in situ polymerization [5]. However, despite their potential, these methods face limitations such as the production of hazardous by-products, need for dedicated vacuum and masking, and difficulty in achieving custom-designed electrodes [5].

In the present work, we introduce a fully additive manufacturing (AM) approach for the fabrication of a CE on polymer parts. The proposed approach sequentially involves the following: (1) rapid prototyping of polymer substrate through 3D printing; (2) cold spray (CS) surface metallization; and (3) deposition of a thin-layer catalyzer. In detail, firstly, polylactic acid (PLA) substrate is accurately produced through material extrusion-based 3D printing. Herein, the 3D printing of polymer substrates offers significant advantages, including rapid prototyping of customized parts, near-net-shape design of intricate components, reduced material waste, and on-demand production [14,15]. Next, the substrate surface undergoes conductive metallization via CS particle deposition, which facilitates the solid-state direct writing of micron-scale metal particles on the polymer surface as a thin-film due to its unique features, including low-process temperature, high adhesion strength, corrosion resistance, and durability [16,17]. In CS deposition, fine metal particles in a size range of 5–50 µm are supersonically (300–1200 m/s) impacted onto a target surface [11]. Under this high-speed impact, the particles undergo intensive plastic deformation, resulting in dense and consolidated functional surface deposition on the target substrate. As such, CS is a solid-state deposition process that offers remarkable advantages for functional metallization on polymeric substrates with minimal disruptive effects of oxidation, thermal residual stress, and phase transformation. Lastly, to create an active catalytic film, a thin layer of graphite is deposited on the as-CS layer with a graphite pencil.

The key and novel contribution of this paper lies in (i) integrating the emerging CS deposition technique into the field of DSSC; and (ii) establishing a fully AM approach that facilitates rapid and sustainable production of polymer-based CEs for DSSCs without the need for environmentally hazardous and cost-intensive processes such as chemical etching, precursor, vacuum, plasma, and vaporization steps.

## 2. Materials and Methods

### 2.1. Methodology

Figure 1b illustrates the proposed AM approach for CEs in DSSCs. It begins with the printing of the polymer substrate using a rapid prototyping technique that relies on material extrusion. The fabrication process involves a typical 3D printer (Sindoh 3DWOX, USA) equipped with a single-nozzle configuration. Polylactic acid (PLA) filament was used as the substrate material due to its recyclability, composability, and low cost [18]. Printing was conducted on a heated bed with a temperature of 90 °C, while the extruder temperature and thickness of each layer were set at 200 °C and 0.2 mm, respectively. Throughout the printing process, the extruder temperature was maintained below the decomposition temperature of the PLA filaments, which is 250 °C [19]. Under these 3D printing configurations, the PLA substrates with dimensions of 25 mm length, 20 mm width, and 3 mm thickness were fabricated. That said, 3D printing of the substrate holds great potential for producing polymer CEs for DSSCs, owing to the critical advantages of 3D printing, including design flexibility, rapid prototyping, reduced material waste, ease of iteration and customization, and cost-effectiveness [14].

**Figure 2:**
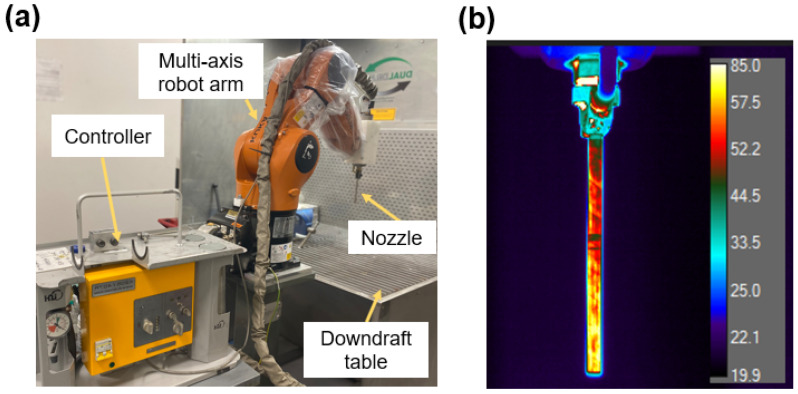
(**a**) Cold spray experimental setup; (**b**) thermal camera image of the nozzle.

Next, the surface of the 3D-printed PLA part is metallized by using CS particle deposition technology. In the present study, the feedstock Tin (Sn) particles with a size range of 10–45 µm [20] were cold deposited on the 3D printed PLA surface through a CS system (Rus Sonic Inc., Model K205/407R, Russia), in which the deposition nozzle was mounted on a programmable robot arm (Kuka KR Agilus). The CS experimental setup is shown in Figure 2a, and further details regarding the experimental setup can be found in the authors’ previous works [21,22,23]. Air was used as the compressed propellant gas at a gauge pressure of 0.7 MPa. The gas flow was preheated through the heating coil of the deposition head, and the temperature of the gas was measured to be nearly 85 °C from the nozzle tip at steady-state conditions by an infrared thermal camera (FLIR A300, New York, NY, USA) (see Figure 2b). The nozzle transverse speed and spray distance were set to 100 mm/s and 10 mm, respectively, by using the robot arm. The CS process settings led to the achievement of well-consolidated and electrically conductive Sn film on the 3D printed PLA parts with a single spray pass.

Subsequently, a thin layer of catalyzer graphite film was over-deposited on the as-CS surface using a graphite pencil (4B), which is often used for low-cost DSSC applications due to its low-cost and good catalytic activity [24]. As such, the proposed manufacturing approach enables the complete AM of CEs by employing sustainable manufacturing practices, offering a green manufacturing solution for DSSCs. The operational settings of each process step are listed in Table 1.

Lastly, the fabricated CEs were sandwiched with the photoanode to construct the DSSCs for performance evaluation. The performance of the DSSCs with the additively manufactured CEs (AM-CE) was compared against the control DSSCs, which employ the traditional FTO/glass-based CEs. Details regarding the fabrication of the DSSCs are presented in Section 3.3.

### 2.2. Characterization Methods

The microstructure of the fabricated electrodes was analyzed by scanning electron microscopy (SEM) (Hitachi S-4800, Japan) equipped with an X-ray (EDX) detector. The average surface roughness (R_a_) of the electrodes was measured by a surface roughness tester (AMTAST, USA). The electrical resistance was measured by a digital multimeter (Agilent/HP 34401A, Melbourne, FL, USA). Ultraviolet-visible spectroscopy (UV-Vis) of the dye (Eosin Y) was characterized by a UV-Vis spectrophotometer (Cary 60). The cyclic voltammeter (CV) tests were conducted by using a potentiostat (SP-200, Bio-Logic Inc., USA) Lastly, the photo-conversion efficiency of the fabricated DSSCs was measured by using a solar simulator (Enlitech SS-F5-3A, USA) with a Keithley 2450 source meter.

## 3. Results and Discussions

### 3.1. Microstructure Analysis

**Figure 3:**
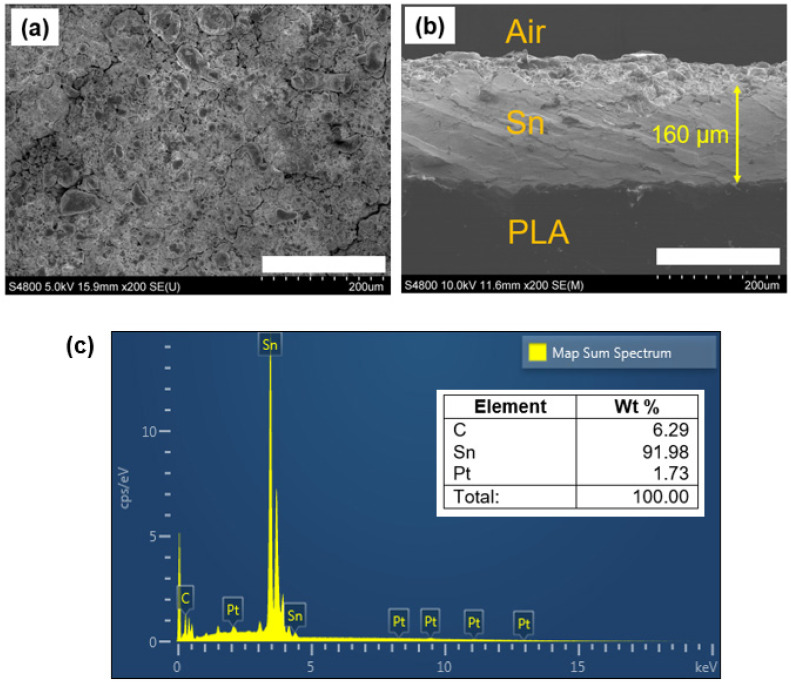
(**a**) Surface; (**b**) cross-section SEM images of the as-CS-metallized PLA sample, (**c**) EDX analysis of the as-CS PLA surface (scale: 200 µm).

Figure 3a,b shows the surface and cross-section SEM images of the as-CS PLA polymer, respectively. As seen in Figure 3a, the Sn surface was successfully deposited on the polymer surface, resulting in a well-consolidated metal (Sn) film with insignificant porosity. Even though cracks were locally observed on the surface, the fabricated surface maintained its high electrical conductivity, indicating continuous junction among the Sn particles. To obtain consistent electrically conductive CS coating on polymers, the metallurgical bonding of the metal microparticles with low porosity and continuous junction is critical [25].

The cross-section morphology in Figure 3b also confirms the metallurgical bonding of the particles into the polymer target. Moreover, the particles were able to impinge into the PLA interface, resulting in a film deposition with a thickness of ≈160 µm. Overall, a dense and consolidated Sn layer was achieved on the polymer surface. Moreover, the EDX analysis in Figure 3c confirms that the Sn surface had a weight ratio of ≈92%. Note that the existence of platinum (Pt) peaks in the EDX analysis is due to the thin-film pre-sputter coating employed to prevent charging effects during the SEM and EDX analyses.

### 3.2. Electrical Characterization

The electrical conductivity of the additively manufactured counter electrode (AM-CE) was calculated by using Equation (1), where σ is the electrical conductivity, *R* is the resistance, and *A* is the cross-sectional area of the circuit. The thickness of the electrode was obtained from the cross-sectional SEM image in Figure 3b. The length, width, and thickness of the electrodes for conductivity calculation are 25 × 10^−3^ m, 20 × 10^−3^ m, and 160 µm, respectively, where the average resistance is 0.092 ohm. Taken together, the electrical conductivity of the polymer electrodes was calculated to be 8.5 × 10^4^ S/m, which is two orders less than the bulk conductivity of Sn (9.17 × 10^6^ S/m), thereby indicating promising electrical conductivity for DSSC applications.

(1) $$\sigma =\frac{L}{RA}$$

**Figure 4:**
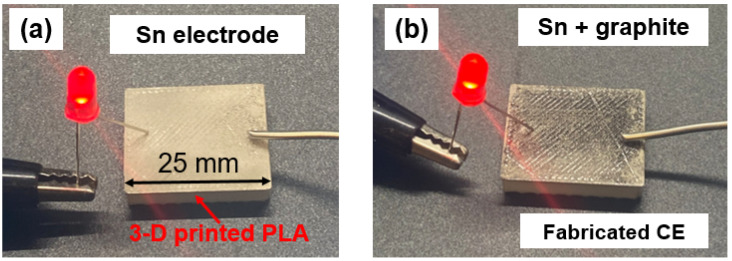
Representative image of the (**a**) as-CS Sn electrode; (**b**) fabricated CE.

For demonstration purposes, a red LED light was connected to the fabricated CE. As seen in Figure 4a,b, the LED light displayed clear illumination on the CE, confirming the electrical conductivity of the fabricated metal film on the 3D-printed polymers. This successful demonstration underscores the feasibility of CS metallization on 3D-printed polymer parts. Additionally, the resulting surface possessed intrinsic surface roughness (6.317 ± 0.87), which is a highly desirable feature in DSSCs. Enhanced surface roughness promotes increased active surface area, facilitating improved charge transfer within the solar cell. Thus, the integration of CS deposition not only ensures electrical conductivity but also enhances surface characteristics, which are crucial for efficient energy conversion.

**Figure 5:**
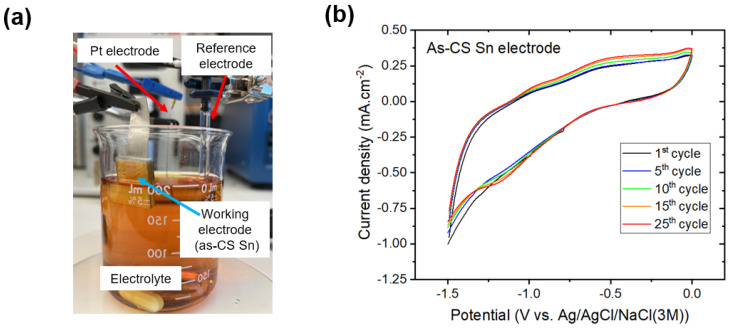
(**a**) CV test setup; (**b**) CV curves of the as-CS Sn electrode under various cycles.

Cyclic voltammetry (CV) tests were also conducted to investigate the electrochemical stability of the as-CS Sn electrode. In these tests, a three-electrode configuration was employed, consisting of the following: (1) the reference electrode (silver/silver chloride (Ag/AgCl)); (2) platinum (Pt) electrode; and (3) as-cold-sprayed Sn (i.e., as-CS Sn) as the working electrode. All the electrodes were cleaned with deionized water prior to the CV tests. The electrolyte was composed of an acetonitrile solution containing 10 mM lithium iodide (LiI), 1 mM iodine (I_2_), and 0.1 M lithium perchlorate (LiClO_4_) [26]. A representative image of the CV setup is shown in Figure 5a.

Figure 5b presents the CV curves of the fabricated as-CS Sn electrode on the 3D-printed PLA part. The voltammetry cycle was set from 0 V to −1.5 V, and the scanning rate was 50 mV/s. As seen in Figure 5b, the Sn electrode exhibits distinct reduction/oxidation curves in each cycle, indicating catalytic activity. Moreover, the current density response of the electrode was maintained without a significant change, showing promising electrochemical stability. As such, the CV tests reveals that the as-CS Sn electrode holds catalytic activity and electrochemical stability, thereby showing the potential to constitute a CE after further applying a thin layer of catalyzer such as graphite film. In that manner, the fully additively manufactured CEs in this study can be a potential candidate for high-performance, stable, low-cost, and green CEs in DSSC technology.

### 3.3. Fabrication of the DSSCs

The DSSCs with the AM-CE and FTO/glass (control) were fabricated. Note that a thin layer of graphite deposition was applied to both substrate materials (as-CS PLA and FTO/glass) enclosing the cell region to create CEs for the performance comparison of fabricated DSSCs. Both CEs had identical dimensions: length = 25 mm, width = 20 mm, and thickness = 3 mm.

#### 3.3.1. Photoanode

**Figure 6:**
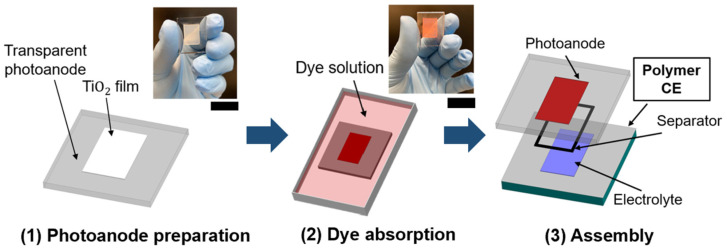
Schematic of DSSC preparation process.

**Figure 7:**
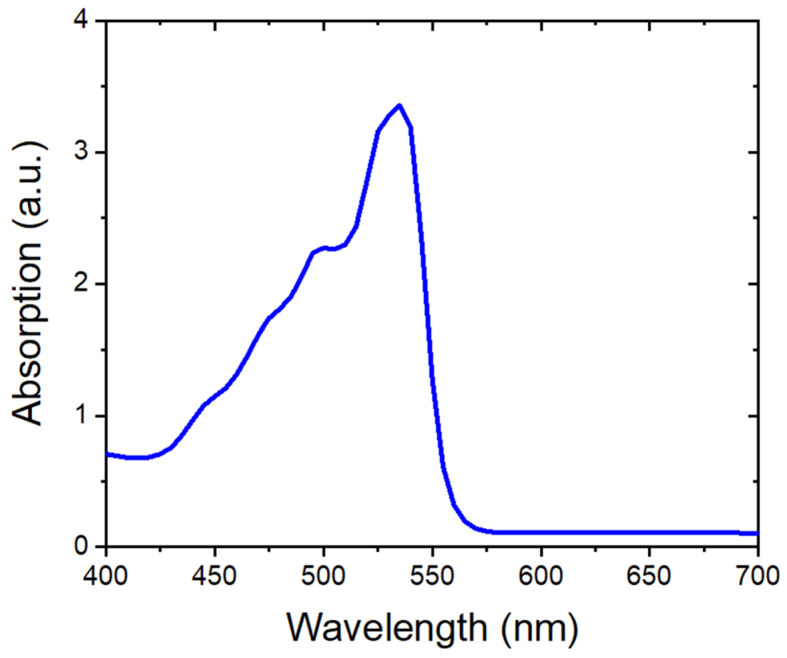
UV-Vis absorption spectra of the Eosin Y dye.

The photoanode of the DSSCs should involve transparent materials to absorb the incident light through the dye-sensitized semiconductor film to the CE. In this regard, FTO/glass was chosen as the photoanode material for all the DSSCs in this study. To prepare the photoanode, titanium dioxide (TiO_2_) paste (Aqua Solutions Inc., USA) was applied to the FTO surface by using the conventional doctor blade method. During this process, the thickness of the film was controlled by using 3M Scotch tape, and the thickness of the coated TiO_2_ film was approximately 7 µm. The TiO_2_ film was then dried in atmospheric conditions for 15 min. Next, the coated TiO_2_ film was annealed at 450 °C for 30 min. At this point, the thin film of mesa-porous TiO_2_ semiconductor film had been achieved on the FTO/glass (see Figure 6 (left panel)). Lastly, the prepared semiconductor TiO_2_ film was soaked in 0.3 mM Eosin-Y dye solution for 24 h in the dark condition and then rinsed with ethanol to remove dye impurities (see Figure 6 (middle panel)). The UV-Vis spectra results in Figure 7 show that the absorption peak of the prepared Eosin-Y dye appeared around 525–530 nm, which matches with previous records [27,28]. As such, the prepared dye can absorb incident light in the visible region.

#### 3.3.2. Electrolyte

**Figure 8:**
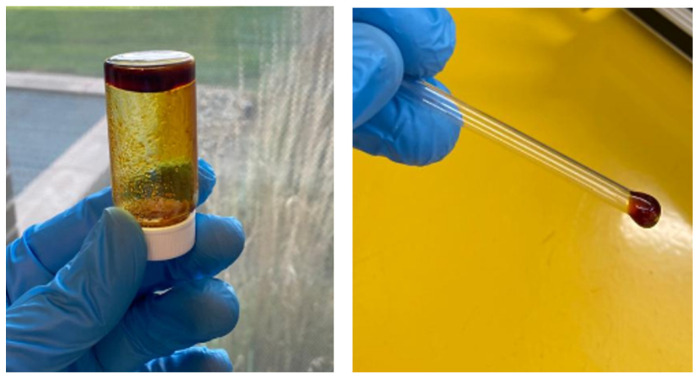
Images of the synthesized quasi-solid gel electrolyte.

A quasi-solid gel electrolyte was used in the fabrication of the DSSCs due to its intrinsic advantages, including improved stability, longevity, and reduced corrosion and leakage [29]. In this regard, a quasi-solid polymer gel electrolyte was synthesized by following a published recipe [30] to prevent any possible electrolyte leak through the porous 3D-printed substrates. Figure 8 shows the representative images of the gel electrolyte.

#### 3.3.3. DSSC Assembly

**Figure 9:**
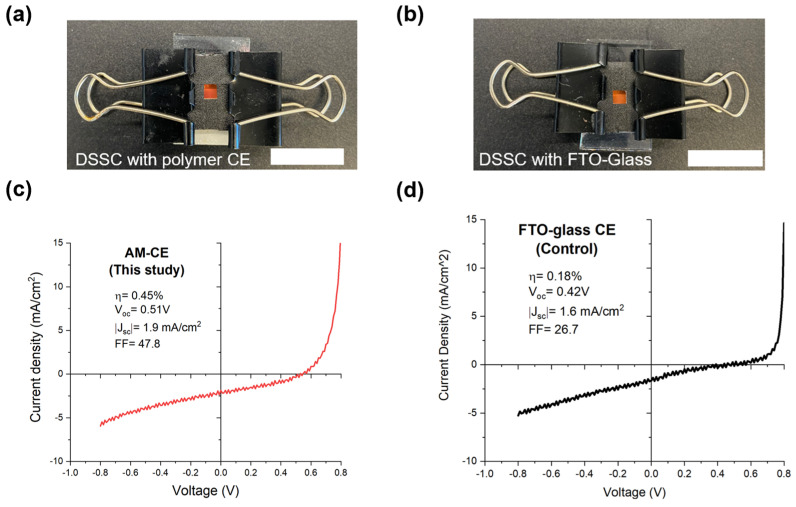
Images of the fabricated DSSCs with (**a**) additively manufactured CE; and (**b**) traditional FTO/glass. Photocurrent-voltage (J-V) curves of the DSSCs with (**c**) polymer CE; (**d**) traditional FTO/glass (control group). Scale bar = 20 mm.

The prepared dye-sensitized photoanode and the fabricated CE were sandwiched together by injecting the synthesized gel electrolyte between the photoanode and the CE (see Figure 6 (right panel)). A thin parafilm (i.e., 130 µm thickness) was used as a spacer between the photoanode and the CE. Slight pressure was applied in the sealing step to help with the penetration of the gel electrolyte into the dye-sensitized semiconductor layer, enabling homogenous wetting of the active cell area. The fabricated DSSCs with the polymer CE and traditional FTO/glass-based CE (control DSSC) are shown in Figure 9a and Figure 9b, respectively.

### 3.4. Performance Evaluation of the DSSCs

The active cell area of the cell was set to 0.16 cm^2^ using a rigid black mask. Current density–voltage (J-V) characterization was carried out under standard illumination conditions with an air mass of 1.5 solar spectra at 100 mW/cm^2^ using a solar simulator [31]. The photoelectric performance criteria of fill factor (FF) and power conversion efficiency (PCE), also known as cell efficiency (η), for the fabricated DSSCs were calculated by using Equations (2) and (3) [32], where V_OC_ is the open-circuit voltage (V), J_SC_ is the short-circuit current density (mA/cm^2^), P_in_ is the incident light power density (mW/cm^2^), and Pmax is the maximum power density (mW/cm^2^) of the cell, respectively.

Note that the efficiency of DSSCs strictly depends on the material selection, including the types of semiconductor film, electrolyte, dye molecules, and catalytic film on the CE. The efficiency of DSSCs that use natural dyes such as Eosin-Y is generally lower (e.g., from η= 0.03% [33] to η= 1.518% [34]) than that of metal-based dyes such as N3-dye (η= 10% [35]) and N719 (η= 11.2% [35]). Moreover, solid-electrolyte gels often lead to lower photo-conversion efficiency as compared to liquid electrodes [36].

For the present study, it is noteworthy that the main objective was not to maximize the efficiency of DSSCs, but rather to conduct a comparative analysis of the performance between the additively manufactured CE and the conventional glass-based CE used in DSSCs, such as graphite-coated FTO/glass. In this regard, the fabricated DSSCs depicted in Figure 9a,b enabled a comprehensive performance comparison between the AM-CE and conventional glass-based CE (FTO/glass) and were fabricated to achieve this.

(2) $$FF=\frac{P_{max}}{V_{OC}\times J_{SC}}$$

(3) $$PCE=\frac{P_{out}}{P_{in}}=\frac{P_{max\;[mW{cm}^{-2}]}}{100}=\frac{V_{OC}\times J_{SC}\times FF}{P_{in}}$$

Figure 9c,d show the J-V characterization results for the DSSCs with AM-CE and FTO/glass, respectively. As seen in Figure 9c,d, the DSSCs with the AM-CE exhibited significantly better efficiency than that of their conventional counterparts with FTO/glass. Table 2 summarizes the J-V results for the DSSCs assembled with different CEs. The results highlight that the DSSCs with the AM-CE can achieve approximately 2.5-times-higher photo-conversion efficiency than the DSSCs with FTO/glass-based CE. Furthermore, the results indicate that the AM-CE enhances catalytic activity, as evidenced by its higher (about 19%) short-circuit current density. It is noteworthy that a low magnitude of J_sc_ was obtained for both CE types, indicating that further optimization of the dye loading, CE materials, electrode/electrolyte interface, and/or fabrication processes may be necessary to enhance the overall efficiency of the solar cells. Therefore, future works may be directed to further characterize the current density by using external quantum efficiency (EQE) tests, across a comprehensive set of wavelengths, and electrochemical impedance spectroscopy (EIS) tests.

The enhanced performance of the DSSCs with the AM-CE can be attributed to the higher electrical conductivity and the micro-rough surface morphology of the AM-CE compared to the traditional counterpart of FTO/glass. Table 3 provides a comparison of the electrical resistance and average surface roughness (R_a_) for both electrodes. As seen in Table 3, the polymer CE offers significantly higher electrical conductivity (lower resistance) and surface roughness than the FTO/glass. This characteristic results in a larger surface area with enhanced electrical conductivity, ensuring a stable charge-carrying capability for the regeneration of dye and consequently leading to improved energy harvesting performance. These proof-of-concept findings suggest that the AM-CE holds potential as a high-performance, cost-effective, facile, and environmentally friendly alternative to the traditional FTO/glass-based CE used in DSSC technology.

It is noteworthy that we observed hysteresis in the fabricated DSSCs, as evidenced by the minor fluctuations along the curves in Figure 9c,d. Hysteresis is a common issue in DSSCs [37], and it is likely attributed to reduced photon absorption, increased charge recombination, and thermal effects [38]. Hysteresis can affect the overall performance of DSSCs by altering their PCE and current J-V characteristics. Addressing hysteresis in DSSCs is essential not only for optimizing device performance but also for ensuring long-term stability and reliability. As such, future work may be directed towards uncovering the exact reasons behind these hysteresis occurrences, as well as to performing stability tests on the solar cells over time.

## 4. Conclusions

A complete AM approach was introduced for fabricating CEs in DSCSS, which sequentially comprises: (1) 3D printing polymer substrates; (2) CS metallization; and (3) pencil graphite deposition. Polymer PLA substrates were printed through material extrusion. Subsequently, the micron-scale Sn particles were then deposited on the as-printed polymer surface by CS at ambient conditions. Lastly, a thin-layer graphite catalyzer was applied to the as-CS surface using a graphite pencil to constitute a fully additively manufactured counter electrode (AM-CE). The following conclusions can be drawn from the present study:

- Unlike traditional CE manufacturing methods, the proposed AM approach eliminates the need for environmentally hazardous and cost-intensive surface pre-treatment processes. This makes it a facile and green approach for CE manufacturing in DSSC technology.
- The resulting AM-CEs exhibited promising electrical conductivity (8.5 × 10^4^ S/m), surface roughness (R_a_ ≈ 6.32 µm), and electrochemical stability.
- Photo-conversion tests confirmed the enhanced performance of AM-CE, exhibiting a ≈2.5-fold increase over the conventional FTO\glass-based CE material in DSSCs.
- The proof-of-concept results underscore the potential of the established complete AM approach for sustainable, large-scale, and low-cost production of the AM-CEs, thereby exhibiting the potential for pragmatic deployment of the DSSC technology.

## Figures and Tables

**Table 1:** Operational settings of each process step.

	Parameter	Setting
(1) 3D printing	Filament diameter (mm)	1.75
	Nozzle diameter (mm)	0.4
	Bed temperature (°C)	60
	Extruder temperature (°C)	200
	Infill density (%)	80
	Layer height (mm)	0.2
	Print speed (mm/s)	40
	First-layer speed (mm/s)	10
(2) Cold spray metallization	Gas type	Air
	Gas pressure (MPa)	0.7
	Powder feed rate (g/s)	0.2
	Nozzle speed (mm/s)	100
	Spray distance (mm)	10
	Number of passes	1
(3) Graphite deposition	A thin film of graphite was over-deposited by a 4B graphite pencil.	

**Table 2:** Summary of J-V characterization.

DSSC with	J_sc_ (mA/cm^2^)	V_oc_ (V)	FF (%)	Efficiency (%)
AM-CE *	1.9	0.51	47.8	0.45
FTO/glass	1.6	0.42	26.7	0.18

* indicates this study.

**Table 3:** Comparison of the counter electrodes.

Material	Electrical Resistance (ohm)	Average Surface Roughness (R_a_) (µm)
AM-CE *	0.092 ± 0.005	6.317 ± 0.87
FTO/glass	36.9 ± 0.535	0.172 ± 0.07

* indicates this study.

## Data Availability

The data supporting the main findings of this study are available from the corresponding authors upon reasonable request.

## Nomenclature

**Symbol**	**Description**
A	Area
FF	Fill factor
J	Current density
J_max_	Maximum short-circuit current density
J_sc_	Short-circuit current density
L	Length
P_in_	Incident light power density
R_a_	Average roughness
R	Resistance
S	Siemens
t	Film thickness
V	Voltage
V_max_	Maximum open-circuit voltage
V_oc_	Open-circuit voltage
ρ	Resistivity
Ω	Ohm
η	Energy harvesting efficiency
**Abbreviations**	**Description**
AM	Additive manufacturing
AM-CE	Additivelt manfuacted counter electrode
CE	Counter electrode
CS	Cold spray
CV	Cyclic voltammetry
DSSC	Dye-sensitized solar cell
EDX	Energy-dispersive X-ray
EQE	External quantum efficiency
FTO	Fluorine-doped tin oxide
ITO	Indium tin oxide
PCE	Power conversion efficiency
PET	Polyethylene terephthalate
Pd	Palladium
PLA	Polylactic acid
SEM	Scanning electron microscopy
Sn	Tin
UV-Vis	Ultraviolet-visible spectroscopy
